# Comparison of video and conventional laryngoscopes for simulated difficult emergency tracheal intubations in the presence of liquids in the airway

**DOI:** 10.1371/journal.pone.0220006

**Published:** 2019-07-19

**Authors:** Kei Suzuki, Shinji Kusunoki, Takuma Sadamori, Yuko Tanabe, Junji Itai, Nobuaki Shime

**Affiliations:** 1 Department of Emergency and Critical Care Medicine, Graduate School of Biomedical and Health Sciences, Hiroshima University, Hiroshima, Japan; 2 Critical Care Medical Center, Hiroshima Prefectural Hospital, Hiroshima, Japan; Imam Abdulrahman Bin Faisal University College of Medicine, SAUDI ARABIA

## Abstract

The presence of vomit, blood, or other foreign liquid materials in the upper airway is a major obstacle in difficult tracheal intubations (TIs) especially in prehospital care. However, the usefulness of video laryngoscopes (VLs) in these situations has not been investigated. The objective of this study was to compare the Airway Scope (AWS) and the Macintosh laryngoscope (ML) for their performance in TIs performed by emergency medical technicians (EMTs) using mannequin models with liquids in the airway. Rice gruel and mock blood were used to fill the upper airways of mannequins to create mock vomit and hematemesis models, respectively. TIs were performed by certified EMTs after visualizing the glottis using an AWS with an 18-Fr suction catheter and a ML with an 18-Fr suction catheter. TIs with AWS and ML were performed in random order in a comparative crossover trial. The TI success rate was evaluated based on the following: (a) the time taken from laryngoscope insertion into the oral cavity to glottis visualization, tracheal tube passage through the glottis, until the initiation of ventilation and (b) the subjective level of difficulty, which was assessed using a visual analog scale (VAS). TIs in vomiting and hematemesis scenarios were performed by 25 and 26 EMTs, respectively. The TI success rates for these scenarios were 100% with both AWS and ML. The median time required until successful ventilation was significantly shorter with AWS than with ML in both the vomiting (42 vs. 58 s) and hematemesis models (33 vs. 39 s), respectively. In the hematemesis scenarios, difficulty assessed using a VAS was lower with AWS than with ML (13 vs. 38 in median), respectively. Compared to the ML, the AWS was capable of faster and easier TIs, in a simulated model of liquid foreign material in the upper airway.

## Introduction

Factors that make tracheal intubation (TI) difficult include cervical spine rigidity, limited mouth opening, obesity, micrognathia, and tongue swelling [1,2]. In TIs performed in emergency outpatient or prehospital care, the presence of liquids or semisolid foreign materials, such as saliva, vomit, or blood in the upper airway increases the difficulty [3–6]. The foreign material blocks the view of the glottis, and the procedure of suctioning prolongs the time taken for the TI. It is particularly important for emergency medical technicians (EMTs) to successfully perform prehospital emergency TI in the presence of liquid or semisolid foreign material.

Video laryngoscopes (VLs) have been shown to be superior to the Macintosh laryngoscopes (MLs) for glottis visualization and have higher TI success rates [7–9]. In the TIs performed in difficult airways reproduced in mannequins [10] and in patients with cervical fixation undergoing elective surgery [11], the Airway Scope (AWS) allows superior glottis visualization and has higher TI success rates than does the ML. However, these studies were performed on mannequins with no foreign materials in the airways and subjects who were undergoing elective surgeries in operating rooms and, therefore, were at little risk of vomiting. The usefulness of the VL in an emergency TI in the presence of saliva, vomit, blood, and other fluids remains to be investigated.

The objective of this study was to compare the usefulness of the AWS and ML in TIs performed by EMTs using mannequin models with the presence of vomit and hematemesis in the airway.

## Materials and methods

This study was approved by the Ethics committee of the Hiroshima University (E-115, E-609). Written consent to participate in the study was obtained from all EMT participants. Data were collected from EMTs who were qualified and certified to perform TIs with ML and AWS in patients with cardiac arrest.

To reproduce scenarios with liquids in the airway, vomit and hematemesis mannequin models were created. The Airway Management Trainer (Laerdal, Stavanger, Norway) was used as the mannequin. Commercially available rice gruel was used as mock vomit, and mock blood used in venous route puncture training was used to simulate hematemesis. The vomit and hematemesis models were created by clamping a mannequin’s trachea and esophagus and then filling the oral cavity up to the incisors with mock vomit or blood.

During the training session, the EMTs received three hours of practical and oral instructions from four specialists in emergency care and anesthesiology on performing TIs with AWS on a regular mannequin, followed by one hour of practical and oral instructions on performing TIs with AWS and ML in a variety of airway obstruction scenarios, including vomiting and hematemesis. Finally, they used an AWS and ML to perform one TI on the vomit or hematemesis mannequin model.

**Fig 1:**
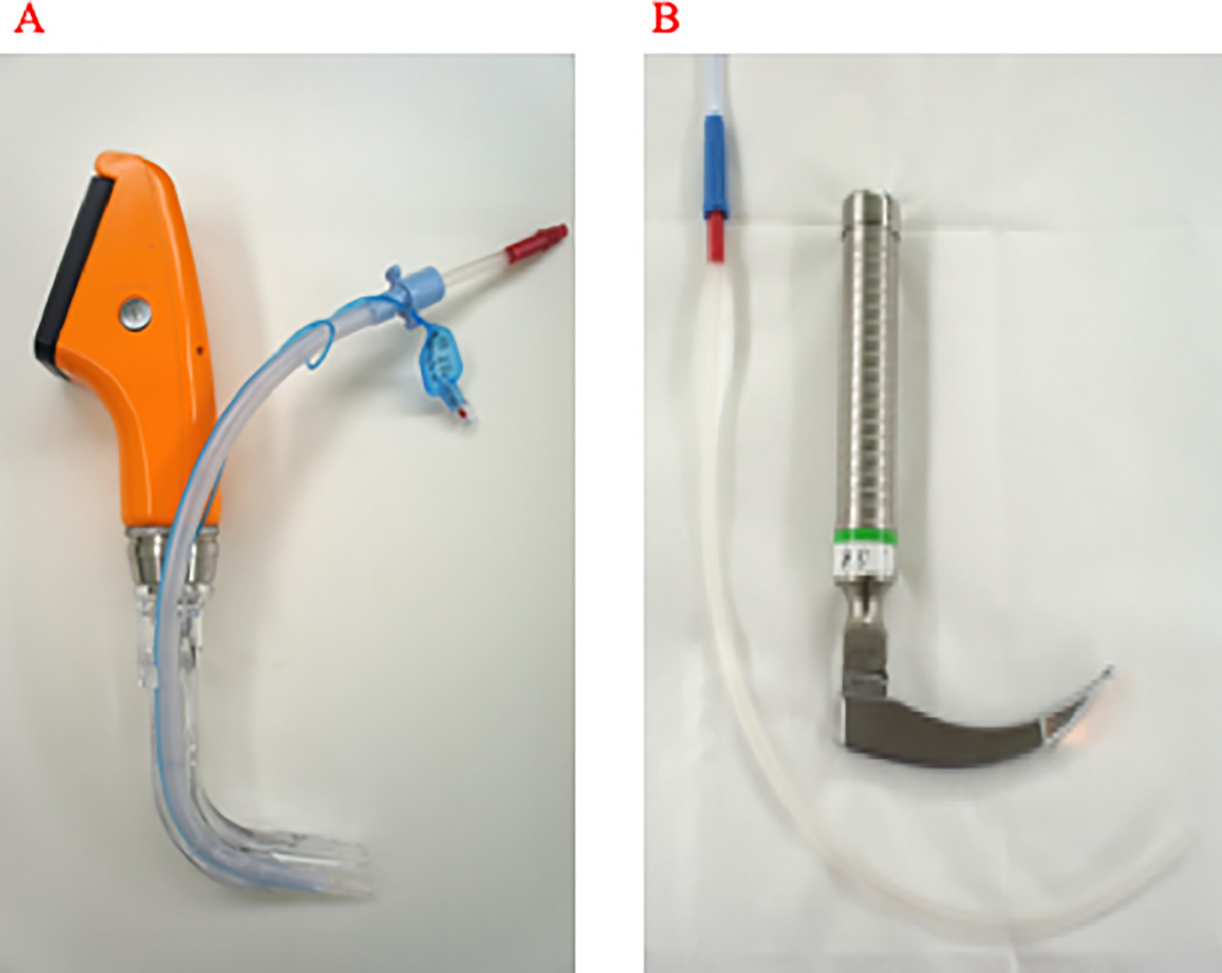
Methods of tracheal intubation and suctioning used in the present study. A. Laryngoscopy with the Airway Scope, which employs suctioning using an 18-Fr suction catheter inserted via the tracheal tube set into the tube-guiding groove of the blade. B. Standard laryngoscopy with a Macintosh laryngoscope with conventional suctioning.

For TIs with an AWS (AWS-S100, Nihon Kohden, Japan), an 18-Fr suction catheter (Terumo, Tokyo, Japan) was first inserted into the tracheal tube (TT) (internal diameter 7.0 mm, Portex, Smiths Medical, Minneapolis, MN, U.S.A.) attached to the guiding groove of the AWS blade (PBLADE, Nihon Kohden, Japan) (Fig 1A). The catheter extended 1 cm deeper than the distal end of the TT. With the suction catheter already connected to the suction device, the AWS was inserted into the oral cavity. The foreign material in the airway was suctioned until the glottis was visualized, and then TI was performed. For TIs with the ML (conventional Macintosh-type blade without external output function; Karl Storz SE & Co., Tuttlingen, Germany), the larynx was exposed using the ML, and the foreign material was suctioned using a suction catheter (18 Fr) (Fig 1B), and the TI was performed after glottis visualization. An assistant EMT aided the primary EMT with the procedure. The suction pressure was set to 20 kPa.

The TI was considered to have failed when the laryngoscope was removed from the oral cavity before completing the TI or when the procedure took ≥ 120 s. Time from the moment of insertion of the laryngoscope into the oral cavity until the completion of suctioning, glottis visualization, tube passage through the glottis, and initiation of bag-valve-mask ventilation were evaluated. Each EMT was required to announce orally when he/she had completed each of the above steps of the TI. The TIs were performed by the EMTs using the AWS and ML in random order to conduct a crossover trial using both methods. All the TIs were video recorded, and emergency care physicians who were not involved in the training sessions used these videos to extract data. A visual analog scale (VAS, 0 mm = very easy, 100 mm = very difficult) was used to subjectively evaluate the level of difficulty in performing the TI with the AWS and ML in both scenarios with liquids present in the airway. Data on the EMTs included their age, experience as a TI-certified EMT, and the number of TIs performed with the ML. Data collection for each scenario was conducted on different dates, by different EMTs and with a different assistant EMT. The study protocol of this study has been disclosed online. http://dx.doi.org/10.17504/protocols.io.3m2gk8e.

A sample size of 25 was estimated as necessary to compare the two laryngoscopes with an initial pass success rate difference of 30%, an α error of 0.05, and a β error of 0.2. The endpoints were TI success rate, the time required for the TI, and difficulty assessed using a VAS. The results were expressed as median values (interquartile range). The TI times and VAS were compared using the Mann-Whitney U test. All analyses were performed using SPSS 23 software (SPSS, Chicago, IL, USA). Statistical significance was accepted at P < 0.05.

## Results

TIs were performed in the vomiting and hematemesis scenarios by 26 and 25 EMTs, respectively. The median age of the participants was 40 years (38–41); they had worked for a median of 10 months (4–35) as TI-certified EMTs and had performed a median of one TI (0–3) with the ML on patients with cardiopulmonary arrest at the site. Only one EMT had used an AWS on an actual patient and had done so six times.

The TI success rates were 100% in both the AWS and ML groups in the vomiting and hematemesis scenarios (Table 1).

**Table 1:** Comparison of the performance of the airway scope and macintosh laryngoscope in vomiting and hematemesis scenarios.

		Airway Scope	Macintosh laryngoscope	*p-value*
Vomiting scenario (n = 26)	Number of successful intubations (%)	26 (100)	26 (100)	-
	Time to visualization of the glottis (sec)	27 (19–34)	41 (28–49)	< 0.01
	Time to intubation (sec)	31 (26–42)	45 (35–56)	< 0.01
	Time to initiation of ventilation (sec)	42 (37–54)	58 (48–70)	< 0.001
	Difficulty (VAS) (mm)	36 (20–50)	46 (25–52)	0.51
Hematemesis scenario (n = 25)	Number of successful intubations (%)	25 (100)	25 (100)	-
	Time to visualization of the glottis (sec)	17 (10–21)	22 (15–34)	< 0.01
	Time to intubation (sec)	21 (16–28)	28 (23–44)	< 0.01
	Time to initiation of ventilation (sec)	33 (25–40)	39 (35–57)	< 0.01
	Difficulty (VAS) (mm)	13 (6–27)	38 (21–53)	< 0.01

VAS: Visual Analog Scale (0 mm = very easy, 100 mm = very difficult).

Data are median (interquartile range).

In the vomiting scenario, time to the visualization of the glottis, termination of intubation, and initiation of ventilation were all significantly shorter with the AWS than with the ML, by 14, 14, and 16 s (median values), respectively (P < 0.01). The VAS scores, however, were not significantly different between the two groups (36 mm in AWS vs. 46 mm in ML) (P = 0.51). Similarly, in the hematemesis scenario time to visualization of the glottis, termination of intubation, and initiation of ventilation were significantly shorter with AWS than with ML, by 5, 7, and 6 s (median values), respectively. The VAS score for the AWS was significantly lower than that for the ML (13 vs. 38 mm, respectively, P < 0.01)

## Discussion

The TIs performed by EMTs on the vomiting and hematemesis mannequin models had 100% success rates using both the AWS and ML. The times from the start of TI to glottis visualization, TT passage through the glottis, and initiation of ventilation were all significantly shorter with the AWS than with the ML. TIs with the AWS were evaluated as subjectively less difficult than those with the ML in the hematemesis scenario.

This was the first study to examine the usefulness of VLs in cases where liquids are present in the airway. The existence and need to suction vomit or blood from the airway and the oral cavity often makes TI difficult and prolonged. “Suction laryngoscopy” has been previously reported to have been used to deal with these situations, which involved attaching a suction port to the ML blade and using this to suction the oral cavity and open the larynx simultaneously [12]. However, while this method reduces the requirement for esophageal intubation, the time taken to perform the TI is comparable with that taken by a regular ML; moreover, it is not widely used, because it is a non-commercial device [12–14].

In the present study, the time from the start of the TI to the initiation of ventilation was about 16 s shorter with AWS than with ML in the vomiting model and about 6 s shorter in the hematemesis model. The differences were primarily due to the reduction in the time taken for glottis visualization. With the AWS, simultaneously performing the suction and intubation manipulations made visualization of the glottis faster. Further, in the method we adopted, the tip of the suction tube was positioned next to the camera, which minimized contamination of the visual field and allowed the suctioning to focus on the glottis. The largest suction tube that can be attached to the groove on the PBLADE is 12 Fr. Inserting an 18-Fr suction tube into the TT may, therefore, have improved the suction efficiency.

A comparison of TI performed with ML for simulated upper airway hemorrhage using regular suction and that with a suction tube pre-inserted into the TT, did not reveal significant differences in the TI success rates or the total time taken [15]. Good glottis visualization provided by a VL [7] could also have contributed to the shortening of the TI times.

Rice gruel and mock blood were used as liquid foreign materials in the present study. The white color and high viscosity of rice gruel may have hindered the indirect visual confirmation of the glottis when the AWS camera was used, explaining why a significant difference in difficulty between the AWS and the ML was not observed in the vomiting scenario. On the other hand, mock blood was deeper and darker in color than rice gruel, interfering with indirect viewing of the glottis physically by VLs. This could be a factor for supporting more advantageous use of AWS in a vomiting scenario than in a hematemesis scenario.

Compared the results of the vomiting and the hematemesis scenario, the time to intubation of both AWS and ML were shorter in the hematemesis than vomiting hematemesis scenario. The data collections of each scenarios were conducted on different dates, by the different EMTs and with the different assistant EMT. So, this difference was not explained by the learning curve effect of multiple training sessions.

In this study, we sought to compare functionality among laryngoscopes using the same flexible suction catheter (18 Fr). Additional studies to evaluate the usefulness of different suctioning devices (including the Yankauer catheter, which is commonly used in clinical situations), should be considered in future research. Ducanto developed the Suction-Assisted Laparoscopy Airway Decontamination (SALAD) technique with a Yankauer catheter when using VLs. SALAD consists of continuous suctioning around the esophageal orifice before the insertion of VLs to deal with continuous regurgitation and to prevent contamination of the camera [16]. Conducting additional studies to assess the efficacy of SALAD by establishing continuous vomiting and hematemesis in mannequin models is also worthwhile.

This study has the following limitations. Liquid foreign materials in the airway encountered in emergency outpatient or prehospital care come in a wide variety of colors, states, and viscosities, including vomit, digestive fluids, food and drink, accidentally swallowed liquids, blood, and phlegm. Thus, results obtained from using mock liquids in the airway may not accurately reflect the actual liquid foreign materials in the airway of a living body. Second, the only VL we examined was the AWS. There are a variety of VL types, and, because the indirect field of view from the camera could be lost partway through the TI in cases of vomiting, an ML-type VL (for example: McGrath MAC, Medtronic Inc., Minneapolis, MN, U.S.A.), which allows both direct and indirect views of the glottis might be useful in such situations. Third, the suction catheter was not pre-inserted in the tracheal tube in the ML group, which could be a biasing factor, as it unavoidably prolongs intubation time. A previous study has however, revealed that this method was not superior to the standard method and regular suction [15]. Additionally, this method is not popular in clinical practice. Fourth, the vomit and hematemesis mannequin models in this study could not reproduce an ongoing regurgitation situation in which cricoid pressure is a recommended response [17]. Fifth, we did not measure the entire time it took to intubate in this study, which can be considered a clinically-important endpoint. Finally, EMTs in Japan are currently only certified to perform Tis in states of cardiopulmonary arrest. In other words, it is highly likely that the TI would need to be performed while chest compressions are being performed. The AWS may be less likely to be influenced by chest compressions than is the ML, suggesting a value in conducting further study using a chest compression model [18, 19].

## Conclusions

The ability of the AWS to visualize the glottis is faster than the ML can shorten the time required to perform TIs in the presence of liquids in the airway due to vomiting and hematemesis.

## Supporting information

S1 Table Time and difficulty of the tracheal intubation in the vomiting scenario. (XLSX)

S2 Table Time and difficulty of the tracheal intubation in the hematemesis scenario. (XLSX)
